# Identification of *Fusarium verticillioides* Resistance Alleles in Three Maize Populations With Teosinte Gene Introgression

**DOI:** 10.3389/fpls.2022.942397

**Published:** 2022-07-14

**Authors:** Xuanjun Feng, Hao Xiong, Dan Zheng, Xiaobing Xin, Xuemei Zhang, Qingjun Wang, Fengkai Wu, Jie Xu, Yanli Lu

**Affiliations:** ^1^State Key Laboratory of Crop Gene Exploration and Utilization in Southwest China, Chengdu, China; ^2^Maize Research Institute, Sichuan Agricultural University, Chengdu, China

**Keywords:** *Fusarium verticillioides*, maize, teosinte, QTL, germplasm resources

## Abstract

Fusarium ear rot (FER) is a common fungal disease in maize (*Zea mays* L.) caused by *Fusarium verticillioides*. Resistant germplasm resources for FER are rare in cultivated maize; however, teosintes (*Z. mays* ssp. *parviglumis* and *Z. mays* ssp. *diploperennis*), which are wild-type species of maize, have the potential to offer a novel source of resistance alleles to enhance pathogen resistance in modern maize. Therefore, the aim of this study was to identify favorable alleles that confer significant levels of resistance toward FER. Three populations of BC_2_F_8_ recombinant inbred lines (RILs) were developed by crossing two different teosintes, *Z. diploperennis* and *Z. parviglumis*, with maize inbred lines B73 and Zheng58, and were screened for FER resistance. We found that *Z. diploperennis* and *Z. parviglumis* had higher resistance toward *F. verticillioides* in the leaves than B73 and Zheng58. However, the resistance toward *F. verticillioide*s in the leaf and ear was unrelated among RILs. FER resistance was positively correlated with grain yield in the B73 × *diploperennis* (BD) and Zheng58 × *parviglumis* (ZP) populations, partly because the quantitative trait loci (QTLs) of FER resistance and yield traits were located close together. Four coincident QTLs (*qFER*bd5.177, *qFER*bd10.140, *qFER*zp4.066, and *qFER*zp5.116) and two highly reliable resistance-yield synergistic QTLs (*qFERbd*10.140 and *qFER*zp4.066) were identified in the BD and ZP populations, opening up the possibility of breeding for FER resistance without reducing yield.

## Introduction

*Fusarium* is a cosmopolitan genus of filamentous ascomycete fungi that includes many agriculturally important plant pathogens (Ma et al., 2013). These fungi can cause serious damage to the roots, stems, leaves, or fruits in different plants, resulting in massive economic losses (Duan et al., 2016). Fusaria also produce a wide range of mycotoxins, such as fumonisins and trichothecenes, which can contaminate agricultural products and render them unsuitable for consumption (Mesterházy et al., 2012; Duan et al., 2016; Lanubile et al., 2017). *Fusarium verticillioides* is the primary cause of Fusarium ear rot (FER) (Logrieco et al., 2002; Folcher et al., 2009; Lanubile et al., 2017). FER causes contamination of grains with polyketide fumonisin mycotoxins, such as fumonisin B1 (FB1), which can cause equine leukoencephalomalacia, porcine pulmonary edema, liver cancer in rats, and neural tube defects in mice (Mesterházy et al., 2012). Furthermore, fumonisins have also been associated with esophageal cancer in humans (Mesterházy et al., 2012). The production of fumonisins by *F. verticillioides* depends on a gene cluster (*FUM*) composed of 16 contiguous and co-expressed genes; deletion of the gene *FUM1* reduces FB1 production by 99% (Glenn et al., 2008). Therefore, breeding for resistance to FER is considered the most environmentally friendly and cost-effective strategy to prevent fumonisin contamination (Mesterházy et al., 2012; Lanubile et al., 2017). At present, the majority of inbred and hybrid maize cultivars are moderately resistant or susceptible to FER (Mesterházy et al., 2012; Mu et al., 2019). Therefore, developing and deploying genetically resistant maize varieties is an efficient strategy for controlling ear rot caused by *Fusarium* spp. and reducing the incidence of fumonisin contamination (Mesterházy et al., 2012).

Fusarium ear rot resistance is under polygenic control and is strongly influenced by environmental factors (Mesterházy et al., 2012; Zila et al., 2013). Although genetic variation toward FER resistance exists among inbred lines and hybrids in field maize, there is no evidence of complete resistance to either ear rot or fumonisin accumulation (Mesterházy et al., 2012). Thus, the identification of genes imparting FER resistance would facilitate their introgression into commercial hybrids. Pérez-Brito et al. identified nine and seven quantitative trait loci (QTLs) in two F_2:3_ populations, respectively, three of which (on chromosomes 3 and 6) coincided in both the populations (Pérez-Brito et al., 2001). In contrast, Robertson-Hoyt et al. identified seven FER-related QTLs and nine fumonisin resistance-related QTLs from two BC_1_F_1:2_ populations, among which three FER-related and two fumonisin-related QTLs were mapped onto similar positions. Furthermore, two QTLs on chromosomes 4 and 5 were present in both populations (Robertson-Hoyt et al., 2006). In an F_2_ mapping population, Kozhukhova et al. discovered a codominant marker, *RGA11*, for FER on the short arm of chromosome 1 at 18.3 cM of the resistance locus (Kozhukhova et al., 2007). Ding et al. investigated FER resistance in a recombinant inbred line (RIL) population of 187 genotypes, among which two QTLs on chromosome 3 were coincident across environments (Ding et al., 2008). Zila et al. identified seven single nucleotide polymorphisms (SNPs) associated with disease resistance in a panel of 1,687 diverse inbred lines. The alleles conferring greater disease resistance at all seven SNPs were rare overall (below 16%) and always higher in allele frequency in tropical maize than in temperate dent maize (Zila et al., 2014). From a population of 818 tropical maize inbred lines, 45 SNPs and 15 haplotypes located within or adjacent to 38 candidate genes were identified to be significantly associated with FER resistance (Chen et al., 2016). Eight loci on chromosomes 2, 3, 4, 5, 9, and 10 were consistent with QTLs from four biparental populations (Chen et al., 2016). Ju et al. identified 8 QTLs and 43 genes that were correlated with *Fusarium* seed rot (FSR) resistance through linkage mapping and genome-wide association study (GWAS), respectively; three loci were detected in both linkage mapping and GWAS (Ju et al., 2017). Maschietto et al. identified 15 QTLs related to FER and 17 QTLs related to fumonisin contamination in an F_2:3_ maize population developed by crossing the CO441 (resistant) and CO354 (susceptible) genotypes (Maschietto et al., 2017). A significant positive correlation was detected between FER and fumonisin contamination (Maschietto et al., 2017). Mu et al. identified 28 genes related to *Fusarium* cob rot in 258 maize inbred lines using GWAS, among which two candidate genes were detected in the previously reported *qRcfv2* region (Mu et al., 2019). Zila et al. (2013) used GWAS to detect allele variants associated with increased FER resistance in a maize core diversity panel of 267 inbred lines evaluated in two sets of environments. However, only three marker loci were found to be significantly associated with disease resistance in at least one subset of environments, and each associated SNP locus had only a minor additive effect on disease resistance (Zila et al., 2013). Overall, a large number of QTLs for FER resistance have been identified in maize, but only a few have been cloned because of their small genetic effect and extreme difficulty in phenotypic evaluation.

The domestication of maize is proposed to have started with the Balsas teosinte (*Zea parviglumis*) ~9,000 years ago in tropical southern Mexico (Matsuoka et al., 2002; Heerwaarden et al., 2011). Long-term breeding of maize has produced a set of desirable traits suitable for human consumption and adapted to the cultivation conditions. However, many teosinte resistance traits to specific environmental conditions, such as edaphic stress and pest pressures, have been lost in modern maize (Mano and Omori, 2007; Burton et al., 2013; Lange et al., 2014; Chen et al., 2015; Wang et al., 2019). This competition between plant growth and resistance is a balancing act to optimize fitness and is called a growth–defense trade-off (Huot et al., 2014). There are limited studies available that truly support the notion that teosintes or maize would be more or less resistant to pathogens (Lange et al., 2014). Nevertheless, as many of the pest resistance mechanisms are also involved in pathogen resistance, it is evident that teosintes have the potential (albeit poorly explored) to reveal traits that can help enhance pathogen resistance in modern maize (Lange et al., 2014). Accordingly, numerous reports have indicated the presence of disease-resistance genes in teosintes. *Z. perennis* is resistant to maize dwarf mosaic virus, maize chlorotic dwarf virus, maize chlorotic mottle virus, and maize streak virus (Nault et al., 1982). *Z. parviglumis* has been reported to be resistant to *Colletotrichum graminicola* (Ces.) Wils. (M1.001) (Lange et al., 2014). Alloplasmic inbred lines from a cross between maize and *Z. diploperennis* exhibited resistance against *Helminthosporium turcium* Pass and *Helminthosporium maydis* Nisik (Wei et al., 2003). Progenies with gray leaf spot (GLS) resistance and resistant QTLs were identified in several populations with teosinte gene introgression in the B73 background (Lennon et al., 2016). Recently, two alleles for resistance to southern leaf blight, northern leaf blight, GLS, and southern corn rust were identified in teosinte (Lennon et al., 2017; Wang et al., 2021).

Given the potential of teosinte germplasm to enhance pathogen resistance in modern maize, *Z. parviglumis* and *Z. diploperennis* were crossed with two popular maize inbred lines, B73 and Zheng58, to generate three populations. The aim of this study was to identify alleles that confer a significant level of resistance to FER in the three populations.

## Materials and Methods

### Construction of Maize–Teosinte Hybrid Populations

B73 and Zheng58, two popular elite inbred maize lines (*Zea mays* L.), were used in this study; *Z. diploperennis* and *Z. parviglumis* were the teosinte species that served as the source of FER resistance germplasm. This process was reported in our previous study (Wang et al., 2019). Briefly, B73 and *Z. diploperennis*, B73 and *Z. parviglumis*, and Zheng58 and *Z. parviglumis* were crossed to obtain maize–teosinte hybrids (F_1_ generation) in 2012. Thereafter, two cycles of backcrossing were performed using B73 and Zheng58 as recurrent parents. The progenies were then self-pollinated seven times and sib-mated to maintain their vigor. Thus, three BC_2_F_8_ recombinant inbred lines (RILs) were obtained, namely, BD (B73 × *diploperennis*), BP (B73 × *parviglumis*), and ZP (Zheng58 × *parviglumis*) populations, with 215, 113, and 122 progenies, respectively.

### Genome Resequencing and Genotyping

Young leaves from each progeny were collected for DNA extraction. The DNA content was measured using a Qubit fluorometer (Invitrogen, USA), and sample integrity and purity were assessed using agarose gel electrophoresis. One microgram of qualified genomic DNA was randomly fragmented to an average size of 300–400 bp using an ultrasonicator (Covaris, USA) for library construction. Genome resequencing was performed by Beijing Genomics Institute Co. Ltd. (Shenzhen, Guangdong, China). Additionally, 150 bp paired-end sequencing was performed on an Illumina Hiseq 4000 with a 10 × average sequencing depth. Raw data were cleaned using the NGS QC Toolkit v2.3.3 with default parameters (Patel and Jain, 2012). Clean reads were aligned to the B73 reference genome (version 4) using the Burrows–Wheeler-Alignment tool (BWA v0.7.13) with default parameters (Li and Durbin, 2009). BAM files were sorted, and PCR duplicates were marked by the SortSam and MarkDuplicates options in Genome Analysis Toolkit (GATK v4.1.2.0), respectively (Mckenna et al., 2010). HaplotypeCaller in GATK was used for SNP calling for each RIL. SNP data for all inbred lines were combined using GenomicsDBImport and genotyped using GenotypeGVCFs in GATK. Furthermore, the GATK tool hard filter was used to filter the variants at the following parameters: MQ < 40.0, DP < 8.0, QUAL < 20, QD < 2.0, ReadPosRankSum < −8.0, FS > 60.0, and MQRankSum < −12.5. Finally, high-quality SNP sets were obtained by filtering with a minor allele frequency threshold of 5% and a missing rate threshold of 20%. High-quality SNPs were prepared for genomic bin construction using Python script SNPbinner (Gonda et al., 2019). All bins, treated as molecular markers, were used to construct a linkage map of the three populations using QTL IciMapping (version 4.2.53). The genetic map is displayed in Supplementary Figure S1.

### Isolation of *F. verticillioides* Race XY-1 and Mycotoxin Content Determination

XY-1 was isolated from diseased maize ears in the field using a single-spore isolation method. The nucleotide sequences of the translation elongation factor 1-α (*EF1*α) and RNA polymerase II largest subunit (*RPB1*) and second largest subunit (*RPB2*) were amplified for sequencing. The nucleotide sequences of primers are listed in Supplementary Table S1. The sequences thus obtained were aligned with sequences in the *Fusarium* database (https://fusarium.mycobank.org/), and XY-1 was identified as *Fusarium verticillioides*. XY-1 was further cultured in solid corn sand medium at 28°C in the dark for 5 days. Then, the contents of fumonisins (FUM), deoxynivalenol (DON), T-2, and zearalenone (ZEN) in the medium were determined using FD-600 (Femdetection, China) *via* fluorescence-based quantitative rapid test strips.

### Evaluation of *F. verticillioides* Resistance in Leaves

The third leaf of B73, Zheng58, *Z. diploperennis*, and *Z. parviglumis* plants at the four-leaf stage, and RIL plants that are 20 days old were used for pathogen inoculation. Six damage points were evenly placed on an 8 cm segment on the middle part of the leaf using a pipette tip, and 2 μl spore suspension (5 × 10^6^ spores/ml) was dropped on each damage point. Detached leaves were floated on water with 1 mg/l of 6-benzylaminopurine (6-BA) in the dark for 4 days and then used for disease index score (DIS) calculation and the maximum quantum yield (QY) measurement. The QY was measured using a FluorCam 800MF (Photon Systems Instruments, Czech Republic) according to the operation manual with the following parameters and protocol: Fv/Fm; Act1: 100%; Super: 90%; Shutter: 1; Sensitivity: 20%. The analyses were conducted using three replicates with nine leaves each. DIS was calculated using the following equation modified from the agricultural industry standard of the People's Republic of China (NY/T1248.8-2016, part 8: Fusarium and gibberella ear rot):

$\text{DIS}=\frac{{\sum LDS}_{i}}{n}$, where *LDS* is the leaf disease score, assigned according to the proportion of diseased area (Table 1); i = 1, 2, 3, 4, …, n; and n is the total number of leaves.

**Table 1:** Classification of leaf disease score.

**LDS**	**Proportion of diseased area**
1	0–5%
3	6–15%
5	16–25%
7	26–50%
9	More than 50%

To determine the biomass of *F. verticillioides* in the inoculated leaves *via* real-time quantitative polymerase chain reaction (qPCR), equal areas of inoculated leaves of each sample were used for DNA extraction. EF1-F1/R1 primers were used to detect XY-1. The primer sequences are listed in Supplementary Table S1.

### Field Evaluations of FER

The three populations were evaluated in 2021 in Chongzhou, China (30° 33′ N, 103° 39′ E) and in 2022 in Xishuangbanna, China (21° 53′ N, 100° 59′ E) with completely randomized trials. Seedlings were planted on 4 April 2021 (Chongzhou), and 3 November 2021 (Xishuangbanna), in 3.5 m single rows with a row width of 0.8 m. Fourteen days after silking for each population, 200 μl spore suspension (5 × 10^6^ spores/ml) was inoculated into the seeds using the side-needle-syringe method, and 20 ears of each row and three replicates of each RIL were inoculated. The FER phenotypes were assessed after the seeds reached maturation using the following equation based on the agricultural industry standard of the People's Republic of China (NY/T1248.8-2016, part 8: Fusarium and gibberella ear rot):

$\text{FER}=\frac{{\sum ERS}_{i}}{n}$, where ear rot score (ERS) was assigned according to the proportion of diseased area (Table 2); i = 1, 2, 3, 4, …, n; and n is the total number of ears (~20) in a replicate.

**Table 2:** Classification of ear disease score.

**ERS**	**Proportion of diseased area**
1	0–1%
3	2–10%
5	11–25%
7	26–50%
9	More than 50%

### Agronomic Traits of Three Populations

Data on different agronomic traits (DTT: day to tasseling, DTA: day to anthesis, DTS: day to silking, ASI: anthesis_silking day, PH: plant height, EH: ear height, REP: ratio of ear height and plant height, EPP: ear number per plant, SD: stem diameter, TBN: tassel branch number, TL: tassel length, EPN: effective plant number, SYPP: standard yield per plant, SPPM: standard production per Mu, SPPH: standard production per hectare, SKW: standard kernel weight per ear, SCW: standard cob weight per ear, SEW: standard ear weight per ear, AKR: average kernel rate, AEL: average ear length, BTL: barren tip length, AED: average ear diameter, RNE: row number per ear, KNR: kernel number per row, KNE: kernel number per ear, KH: kernel height, ACD: average cob diameter, SHKW: standard hundred kernel weight, AWC: average water content, STW: standard test weight, KL: kernel length, KW: kernel width, SOC: standard oil content, SPC: standard protein content, SSC: standard starch content, SLC: standard lysine content, SGC: standard glutamate content) were collected in 2017 from Xishuangbanna and Hainan (18° 10′ N, 109° 11′ E) and in 2018 from Hainan, Chongzhou, and Xishuangbanna. Mu in “standard production per Mu” is a unit used in China. One hectare is equal to 15 Mu. Three replicates were analyzed at all the locations. The best linear unbiased prediction (BLUP) values for each trait at the five locations were used in this study. Partial data from the ZP population were used in our previous report (Wang et al., 2019).

### Quantitative Trait Locus Analysis

QTL IciMapping (version 4.2.53) was used for the QTL analysis with the following parameters: mapping population type: P1BC2RIL; missing phenotype: deletion; mapping with ICIM-ADD (inclusive composite interval mapping for additive/dominant effect) method: step = 0.1 cM, PIN = 0.001, LOD = 2.5; mapping with ICIM-EPI (inclusive composite interval mapping for epistatic interacted effect) method: step = 1 cM, LOD = 5, PIN = 0.0001. Given that epistatic QTL is complex and difficult to apply, ICIM-ADD was mainly used to identify additive QTLs in this study. Given the size of populations, the QTLs from two environments of the BD, BP, and ZP populations with genetic distances of ~10, 20, and 20 cM, respectively, were considered to be a coincident QTL.

### Expression Analysis of Candidate Genes

The leaves of living 14-day-old plants were used for inoculation. Two microliter spore suspension (5 × 10^6^ spores/ml) was dropped on a damage point of leaves and covered with a plastic wrap to keep a relative high moisture condition. The same inoculation was performed using sterile water at the same time points corresponding to XY-1 inoculation and it was used as the control. The expression level of candidate gene under XY-1 treatment was normalized using the control of each point. *ZmGAPDH1* and *ZmeF1*α were used as reference genes to normalize the expression of candidate gene. The primers used are listed in Supplementary Table S1.

### Data Analyses

The analysis of variance and heritability for FER was performed using the R software (R Core Team, 2021). The mean values of DIS and QY from three replicates and the BLUP values of FER from six replicates of each population were used to analyze their correlation. The R packages lme4 (Bates et al., 2015) and lmerTest (Kuznetsova et al., 2017) were used to calculate variance, heritability, and BLUP values for all the samples. Correlation tests of different traits and visualization were performed using ggplot2 (Wickham et al., 2016), corrplot (Wei and Simko, 2021), vcd (Meyer et al., 2006), and psych (Revelle, 2017). The difference in the extent of FER between the two alleles of a QTL was analyzed using Student's *t*-test. Additionally, the genotypic and phenotypic data of three populations are organized into a format suitable for the QTL IciMapping software and displayed in Supplementary Tables S2–S4.

## Results

### Comparison the Leaf Resistance to *F. verticillioides* Between Modern Maize and Teosintes

**Figure 1:**
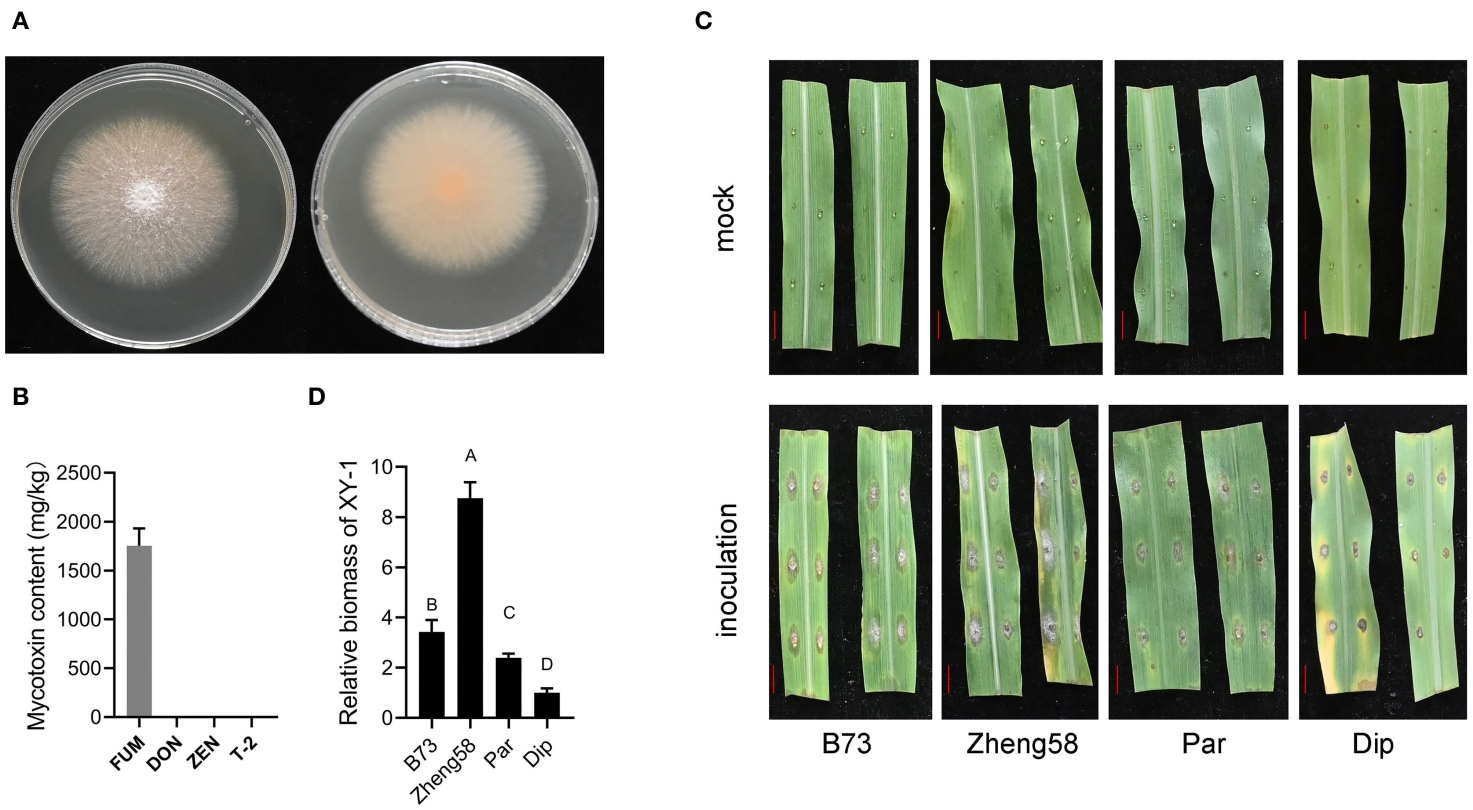
The leaf resistance to *F. verticillioides* between modern maize and teosintes. **(A)** The front and back faces of *F. verticillioides* XY-1 grown on PDA solid medium for 5 days. **(B)** The contents of four mycotoxin produced by XY-1 grown in solid corn sand medium at 28°C in the dark for 5 days. FUM, fumonisins; DON, deoxynivalenol; ZEN, zearalenone. **(C)** Phenotype of detached leaves with and without XY-1 inoculation. The red bar represents 1 cm length. **(D)** The relative biomass of XY-1 in inoculated leaves. Statistical analysis was performed using one-way ANOVA. Different letters on bar represent that the difference was significant at the level of *p* = 0.01. Dip, *diploperennis*; Par, *parviglumis*.

Mycelia of XY-1 appeared white to pale yellow after 3 days of inoculation on potato dextrose agar (PDA) medium (Figure 1A). XY-1 was subsequently inoculated in corn sand medium, and the content of FUM, DON, T-2, and ZEN was determined. Our results revealed that XY-1 produced FUM, a common *F. verticillioides* mycotoxin; however, it did not produce DON, T-2, or ZEN (Figure 1B). Owing to the large differences in ears and seeds between maize and teosinte, the response to *F. verticillioides* infection was tested on the leaves of B73, Zheng58, *Z. diploperennis*, and *Z. parviglumis*. Based on the visible phenotype and biomass of XY-1 in the inoculated leaves of the four samples, we propose that leaf resistance to *F. verticillioides* is the highest in *Z. diploperennis* and lowest in Zheng58 (Figures 1C,D).

### Introgression of Teosinte Genes Into Maize Improves the Resistance to FER

**Figure 2:**
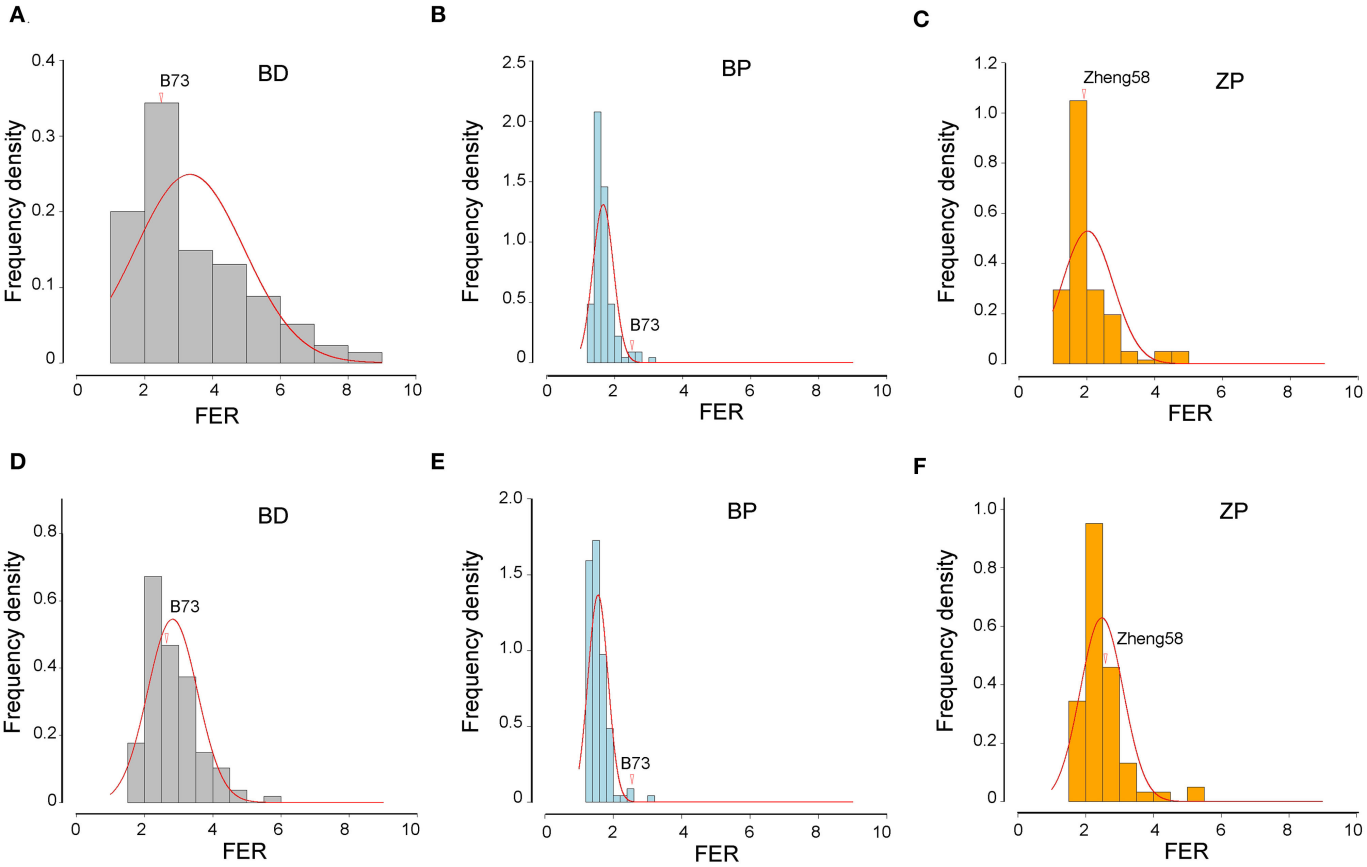
Frequency distribution of FER in three populations. **(A–C)** The data of 2021 in Chongzhou. **(D–F)** The data of 2022 in Xishuangbanna. The vertical axis shows the frequency density. The horizontal axis represents the FER. Frequency = “frequency density” × “FER spacing.” Red lines represent normal distribution fitting curve of each population. The FER of maize parents are indicated by red arrows. The BLUP value of FER from three replicates of each environment was used here.

Three BC_2_F_8_ populations, developed by crossing *Z. diploperennis* or *Z. parviglumis* with maize inbred lines B73 or Zheng58, were evaluated for FER resistance in two field trials over 2 years using side-needle-syringe inoculation of *F. verticillioides* XY-1. We found that the variation amplitude of FER was large in the BD and ZP populations but small in the BP population (Table 3; Figure 2). Furthermore, FER was markedly influenced by environmental effects, and heritability was lower in Xishuangbanna than in Chongzhou for all the three populations (Table 3). Based on the BLUP values of six replicates of each population in two environments, more than 41% of the BD progenies, 95% of the BP progenies, and 86% of the ZP progenies had higher resistance than the corresponding maize parents (Figure 2). Therefore, the introgression of teosinte genes into maize improves FER resistance.

**Table 3:** Descriptive statistics for FER resistance for three teosinte gene introgression populations.

**Population**	**Environment**	**Mean**	**SD**	**CV(%)**	**Range**	**H^**2**^**	$\sigma_{GE}^{2}$	$\sigma_{G}^{2}$
BD	CZ	3.34	1.60	47.9	1.39–8.26	0.89	2.9008	–
	XSBN	2.82	0.73	25.9	1.72–5.84	0.77	0.8476	–
	Combine	3.08	0.87	28.2	1.92–6.49	0.66	1.1462	0.6836
BP	CZ	1.67	0.30	18.0	1.25–3.04	0.63	0.1487	–
	XSBN	1.56	0.29	18.6	1.22–3.19	0.61	0.1403	–
	Combine	1.61	0.13	8.1	1.41–2.32	0.36	0.0507	0.0859
ZP	CZ	2.02	0.75	37.1	1.29–4.88	0.81	0.7067	–
	XSBN	2.48	0.64	25.8	1.75–5.33	0.66	0.6780	–
	Combine	2.20	0.65	29.5	1.50–4.99	0.76	0.5753	0.0499

*CV, coefficient of variation; H^2^, Broad-sense heritability; $\sigma_{\text{G}}^{2}$, genetic variance; $\sigma_{\text{GE}}^{2}$, genetic-environmental interaction variance; CZ, Chongzhou; XSBN, Xishuangbanna*.

### No Correlation Between Leaf and Ear for *F. verticillioides* Resistance

**Figure 3:**
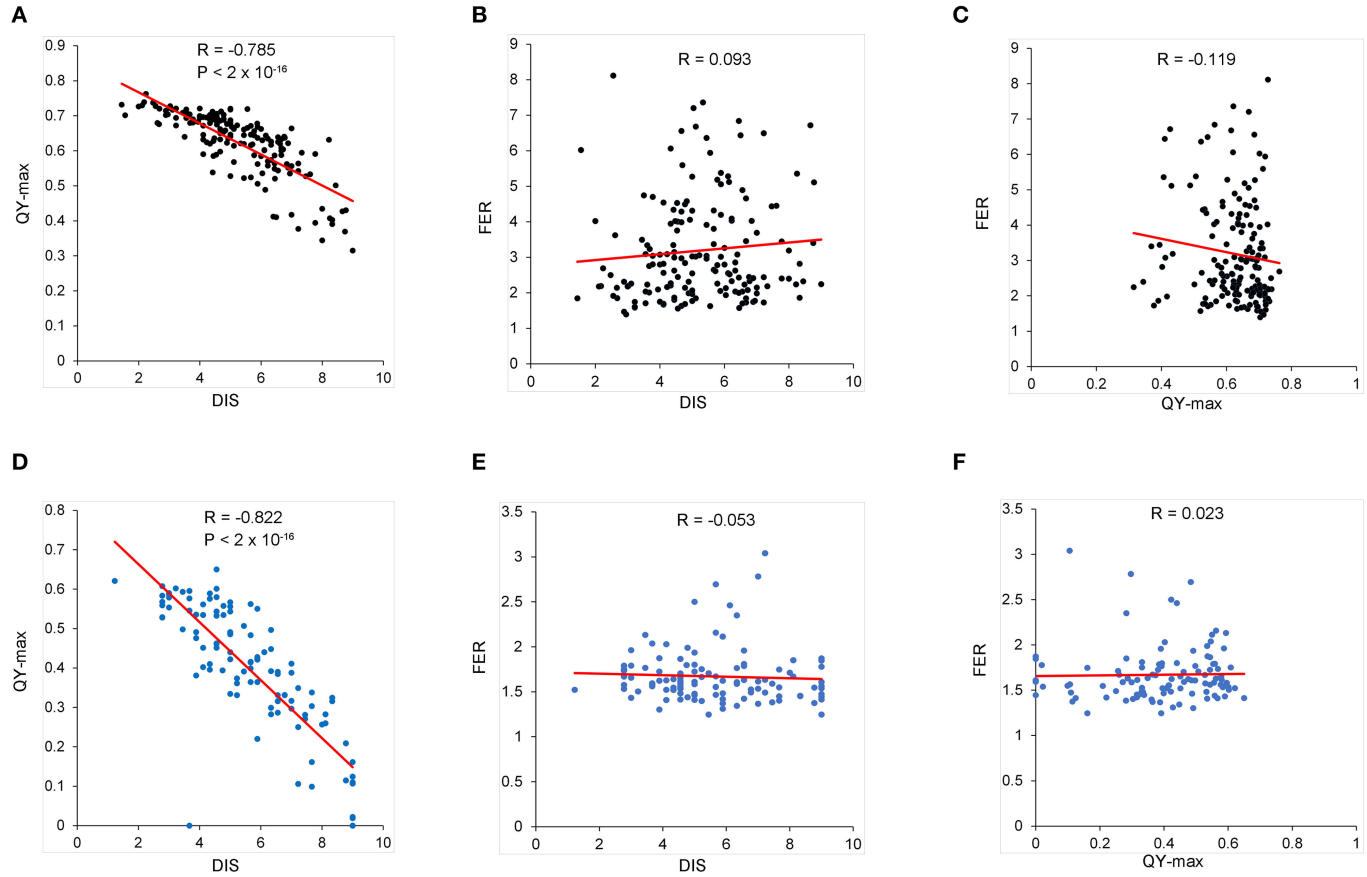
The pairwise correlation among QY, FER, and DIS. **(A–C)** The data of BD population. **(D–F)** The data of BP population. The mean of QY and DIS calculated from three replicates, each with nine leaves, was used here. The BLUP value of FER from six replicates in two environments was used here.

Variations in resistance with age and between tissues are common in plant–pathogen interactions (Develey-Rivière and Galiana, 2007). To determine whether leaf resistance is consistent between the ear resistance, *F. verticillioides* resistance was investigated on the leaves of the BD and BP populations using DIS and QY. We analyzed pairwise correlations among the three parameters (leaf DIS, QY, and FER) in the BD and BP populations. QY value decreased under *F. verticillioides* infection, and it was negatively correlated with DIS with the related coefficients of −0.79 and −0.82 in the BD and BP populations, respectively (Figures 3A,D). However, we found no significant correlation between the FER and DIS of leaves or QY in the two populations (Figures 3B,C,E,F). Thus, *F. verticillioides* resistance was not correlated between the vegetative organs and the ear.

### Correlation Between FER Resistance and Agronomic Traits

**Figure 4:**
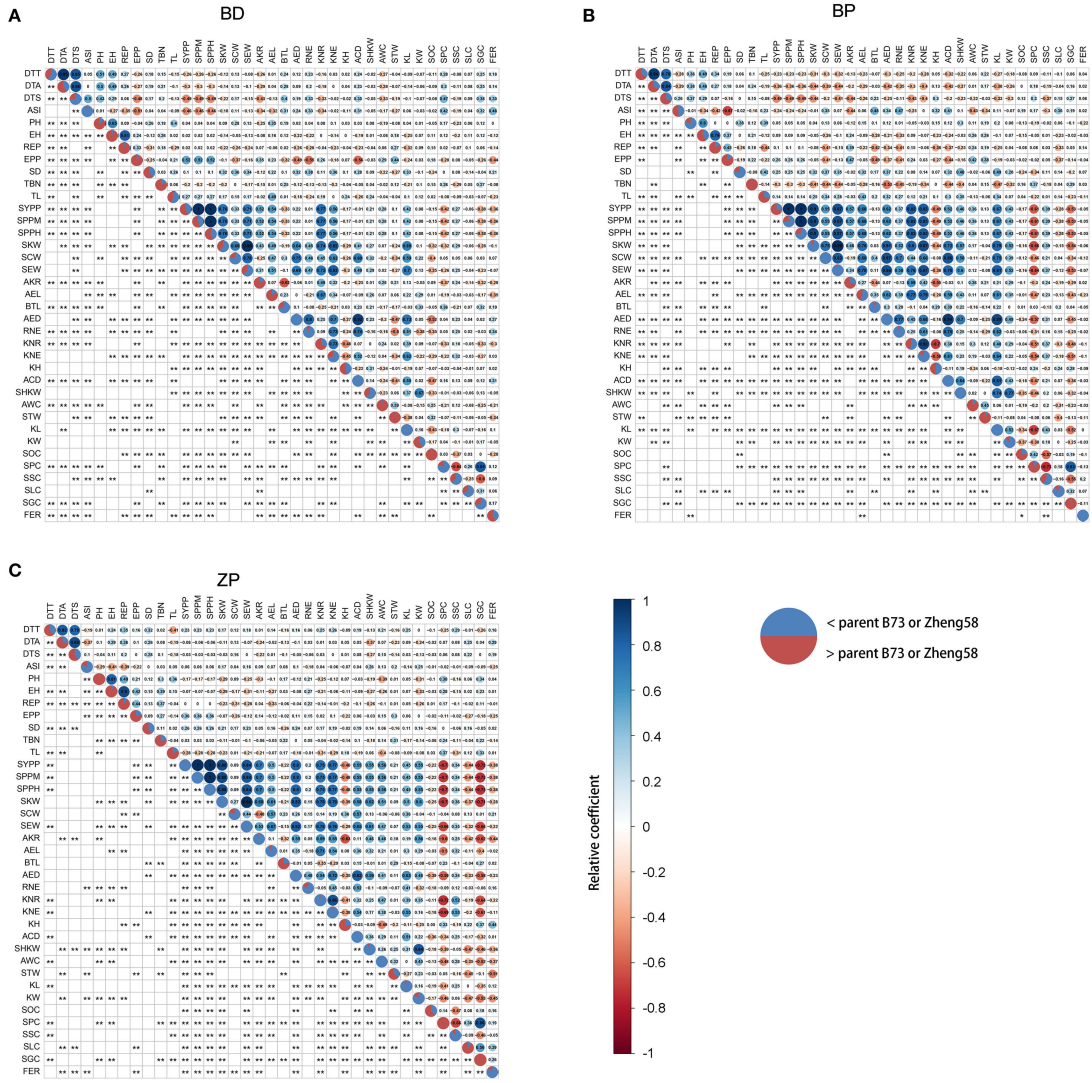
**(A–C)** Correlation between FER resistance and other agronomic traits. The BLUP value of FER from six replicates in two environments and BLUP values of agronomic traits from 15 replicates in five environments were used here. The relative coefficient was represented by the area and color of filled circle labeled from dark red to dark blue in the right upper part of a panel, and the numerical value of the relative coefficient was displayed in the filled circle. Significance was displayed as an asterisk in the left lower part of a panel. *p < 0.05, **p < 0.01. Pie charts on the diagonal were used to show the proportion of progeny with corresponding traits higher (red) or lower (blue) than their maize parents. DTT, day to tasseling; DTA, day to anthesis; DTS, day to silking; ASI, anthesis_silking day; PH, plant height; EH, ear height; REP, ratio of ear height and plant height; EPP, ear number per plant; SD, stem diameter; TBN, tassel branch number; TL, tassel length; SYPP, standard yield per plant; SPPM, standard production per Mu; SPPH, standard production per hectare; SKW, standard kernel weight per ear; SCW, standard cob weight per ear; SEW, standard ear weight per ear; AKR, average kernel rate; AEL, average ear length; BTL, barren tip length; AED, average ear diameter; RNE, row number per ear; KNR, kernel number per row; KNE, kernel number per ear; KH, kernel height; ACD, average cob diameter; SHKW, standard hundred kernel weight; AWC, average water content; STW, standard test weight; KL, kernel length; KW, kernel width; SOC, standard oil content; SPC, standard protein content; SSC, standard starch content; SLC, standard lysine content; SGC, standard glutamate content.

Data from the 2021 field reports revealed that FER resistance (a milder FER phenotype was associated with better resistance) was positively correlated with grain yield (Supplementary Figure S2A); however, we were unable to find similar reports from previous studies. We suspected that this might be because the proportion of diseased area was used to calculate FER. Therefore, the absolute diseased area for the largest population (BD) was used to calculate FER. However, FER continued to show a positive correlation with grain yield; furthermore, its correlation with other agronomic traits did not change (Supplementary Figure S2B). Therefore, the proportion of diseased area was further used for FER calculation. BLUP values of FER from six replicates at two locations also revealed that FER resistance was positively correlated with grain yield in the BD and ZP populations (Figures 4A,C). Additionally, FER was correlated with many other agronomic traits in the BD and ZP populations but with only a few traits in the BP population (Figure 4). We suggest that this is partly because the variation amplitude of FER in the BP population was small (Figures 2B,E). Considering the BD and ZP populations comprehensively, DTA and DTS were positively correlated with FER (Figures 4A,C), whereas EPP, SYPP, SPPM, SPPH, AKR, KNR, AWC, and STW were negatively correlated with FER (Figures 4A,C).

### Deciphering the FER Resistance Loci and Loci Determining FER-Correlated Traits in Three Teosinte Gene Introgressive Populations

**Figure 5:**
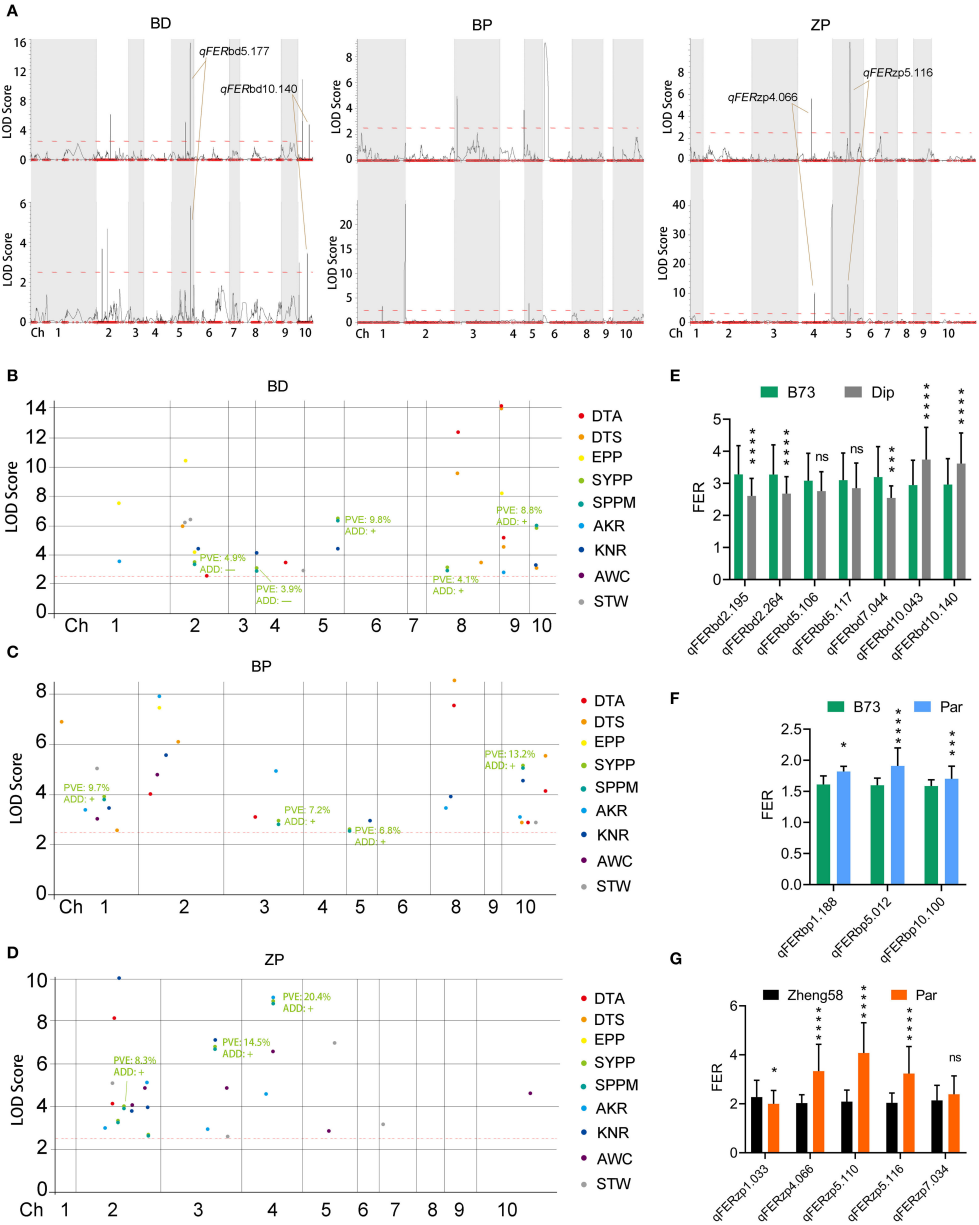
Mapping for additive QTLs for FER and FER-correlated traits. **(A)** QTLs of FER in three populations from 2021 (the upper panel) and 2022 (the lower panel). **(B–D)** QTLs of nine FER-correlated traits in three populations. The BLUP values of each trait from 15 replicates in five environments were used for QTL mapping. The red dotted lines indicate the LOD threshold, and QTLs with higher LOD than this threshold are considered as candidate QTLs. DTA, day to anthesis; DTS, day to silking; EPP, ear number per plant; SYPP, standard yield per plant; SPPM, standard production per Mu; AKR, average kernel rate; KNR, kernel number per row; AWC: average water content; STW: standard test weight. PVE, phenotypic variation explained by this QTL. ADD, the estimated additive effect of maize parent at this QTL. **(E–G)** The difference of FER between two homozygous genotypes of each QTLs. The BLUP value of FER from six replicates in two environments was used for QTL mapping. Both left and right markers were used for difference calculation; and the one with the bigger difference was used to represent the QTL. Dip, *diploperennis*; Par, *parviglumis*. Statistical analysis was performed using Student's *t*-test. Asterisks on bar represent the difference is significant (*p < 0.05, **p < 0.01, ***p < 0.001, ****p < 0.0001). “ns” on bar represents the difference is not significant.

Fusarium ear rot data from two locations were used for QTL mapping using QTL IciMapping with the ICIM-ADD method. In total, 22 *qFER* (QTL for FER) were identified in the three populations from two locations (Figure 5A), in which four *qFER* were identified as coincident QTLs in the BD and ZP populations (Figure 5A; Supplementary Table S5).

SYPP, SPPM, and other FER-correlated traits were used for QTL mapping to further reveal the underlying mechanism for the correlation between FER and other agronomic traits in the BD and ZP populations. We found that two and one *qYield* (QTL for SYPP and SPPM) with the highest phenotypic variation explained (PVE) were located closely to *qFER* in the BD (*qFER*bd5.177 and *qFERbd*10.140) and ZP (*qFER*zp4.066) populations, respectively (Figures 5A,B,D; Supplementary Table S5). Interestingly, the additive effect of maize parents on FER and yield was contrary to these three QTLs (Figures 5B,D; Supplementary Table S5). However, we did not observe a close relationship between *qYield* and *qFER* in the BP population (Figures 5A,C; Supplementary Table S5). Thus, we speculate that the positive correlation between FER resistance and yield in BD and ZP was due to the linkage between *qYield* and *qFER*, and the effect of the same allele on these two traits was consistent. Additionally, we found a similar relationship between *qFER* and *qKNR* (QTL for KNR) in the BP and ZP populations, between *qFER* and *qSTW* (QTL for STW) in the BP and ZP populations, between *qFER* and *qEPP* (QTL for EPP) in the BD population, between *qFER* and *qAKR* (QTL for AKR) in the ZP population, and between *qFER* and *qAWC* (QTL for AWC) in the ZP population (Supplementary Figures S3A,C). However, we did not find any association between *qFER* and *qDTA* (QTL for DTA) or *qDTS* (QTL for DTS) (Supplementary Figures S3A–C).

**Figure 6:**
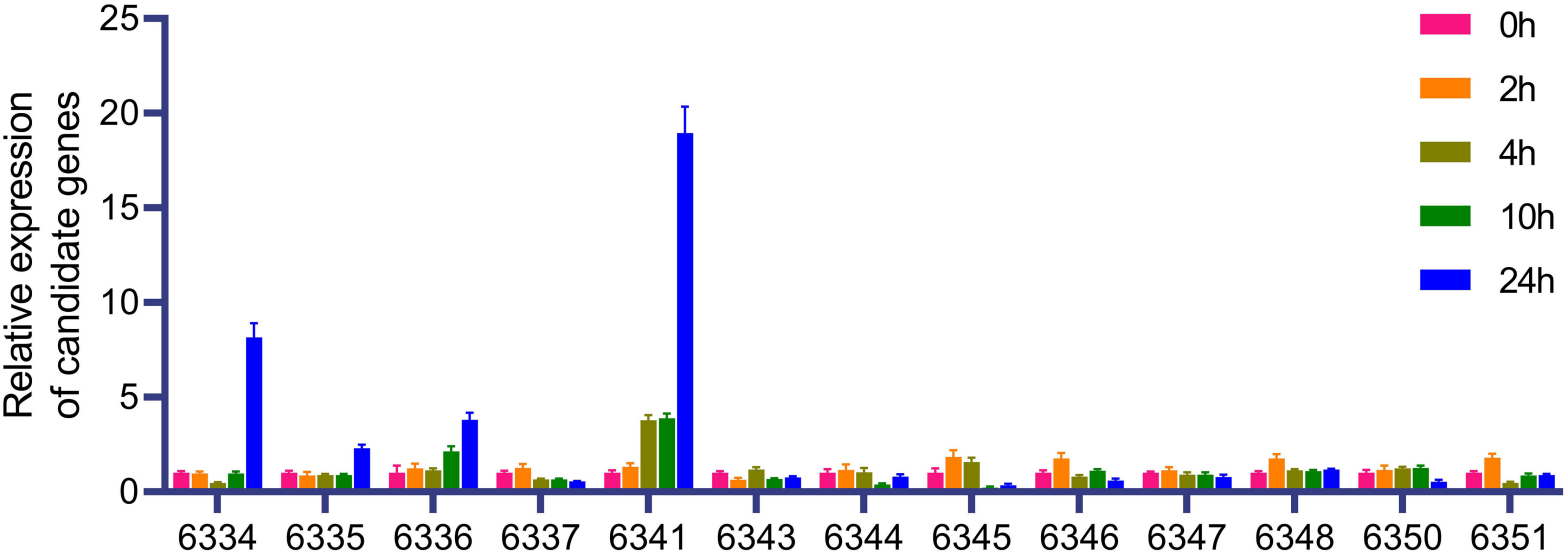
Expression of candidate gene under XY-1 treatment. The last four numbers of the gene number are used to represent the corresponding gene. Complete gene numbers are shown in Supplementary Table S6.

Based on the BLUP value of FER from six replicates at two locations, seven, three, and five QTLs were identified in the BD, BP, and ZP populations, respectively (Table 4), along with the four abovementioned coincident QTLs (*qFER*bd5.177, *qFER*bd10.140, *qFER*zp4.066, and *qFER*zp5.116) (Table 4; Supplementary Table S5). Four epistatic QTLs were identified in the BP population but were absent in the BD and ZP populations (Supplementary Figure S4). To confirm the estimated additive effect of the alleles at each QTL, the FER data were compared between the maize allele and teosinte allele at each QTL. Significant additive effect estimates of the alleles at each QTL were confirmed in 12 of the 15 cases (Figures 5E–G; Table 4). Collectively, *qFER*bd10.140, *qFER*zp4.066, and *qFER*zp5.116 were the most reliable QTLs for FER resistance in the BP and ZP populations. *qFER*bd10.140 and *qFER*zp4.066 were highly reliable, resistance-yield synergistic QTLs. Based on the reference genome, there are 14 genes in *qFER*bd10.140, among which four genes, namely, *Polyamine Oxidase 3* (*PAO3), Autophagy 8b* (*ATG8b)*, a polyphenol oxidase, and an unknown gene, were induced by XY-1 (Figure 6; Supplementary Table S6), and one gene, namely, *Starch Synthase 3* (*SS3)*, was directly associated with plant yield. The regions of *qFER*zp4.066 and *qFER*zp5.116 were large and contained a large number of genes (Supplementary Table S6).

**Table 4:** Significant QTLs for FER in three populations based on data collected in 2 years.

**Population**	**QTL**	**Chromosome**	**LeftMarker**	**RightMarker**	**Position (cM)**	**LOD**	**PVE(%)**	**Add^¶^**	**Confirmed^§^**
BD	*qFER*bd2.195	2	Bin2.195	Bin2.196	66.9995	5.4345	5.8601	0.2211	Yes
	*qFER*bd2.264	2	Bin2.263	Bin2.264	85.7993	3.456	3.6482	0.1738	Yes
	*qFER*bd5.106	5	Bin5.105	Bin5.106	82.5993	6.1512	6.6578	0.3747	No
	*qFER*bd5.177	5	Bin5.177	Bin5.178	113.2988	17.784	22.354	−0.5373	No
	*qFER*bd7.044	7	Bin7.043	Bin7.044	25.8001	2.6835	2.7974	0.183	Yes
	*qFER*bd10.043	10	Bin10.042	Bin10.043	22.8001	8.5171	9.4607	−0.3545	Yes
	*qFER*bd10.140	10	Bin10.140	Bin10.141	66.8996	4.5186	4.8913	−0.2356	Yes
BP	*qFER*bp1.188	1	Bin1.188	Bin1.189	185.8021	3.6376	13.59	−0.1157	Yes
	*qFER*bp5.012	5	Bin5.011	Bin5.012	11.5000	3.567	13.1842	−0.1141	Yes
	*qFER*bp10.100	10	Bin10.099	Bin10.100	88.9992	3.2131	11.7093	−0.0494	Yes
ZP	*qFER*zp1.033	1	Bin_1.032	Bin_1.033	20.0000	3.5559	3.2731	0.1583	Yes
	*qFER*zp4.066	4	Bin_4.065	Bin_4.066	86.3993	20.1623	26.009	−0.5481	Yes
	*qFER*zp5.110	5	Bin_5.109	Bin_5.110	79.0994	19.7373	25.4946	−0.8188	Yes
	*qFER*zp5.116	5	Bin_5.116	Bin_5.117	91.5992	4.5007	5.0696	−0.3671	Yes
	*qFER*zp7.034	7	Bin_7.033	Bin_7.034	25.6001	2.7452	2.4907	−0.1412	Yes

*The grey highlighted loci are coincident QTLs identified from two environments*.

¶ *Positive effects refer that B73 (in the BD and BP population) or Zheng58 (in the ZP population) allele increases FER, and negative effects refer that B73 or Zheng58 allele decreases FER*.

§ *FER data were compared between the maize allele and teosinte allele at each QTL. If the difference of FER is significant and consistent with the Add, the corresponding QTL is denoted as confirmed*.

## Discussion

Teosintes, the wild relatives of maize, are resistant to many environmental stresses, particularly to pests (Lange et al., 2014). However, studies regarding the difference in disease resistance between maize and teosintes are limited (Lange et al., 2014). As ear rot resistance germplasm resources are rare in cultivated maize, we aimed to compare the *F. verticillioides* resistance between teosintes and maize and identify the FER resistance alleles in three populations with teosinte gene introgression.

Given the big difference in ears and seeds between maize and teosintes, leaves were used to compare the *F. verticillioides* resistance between teosinte and maize in this study. *F. verticillioides* and its toxin fumonisin can disrupt sphingolipid metabolism, elicit hypersensitive responses, and destroy the photosynthetic membrane structure in plants (Shi et al., 2007). Therefore, the QY value decreased under *F. verticillioides* infection (Figures 3A,D). The leaves of *Z. diploperennis* and *Z. parviglumis* were significantly more resistant than those of maize cultivars B73 and Zheng58, and *Z. diploperennis* performed the best (Figures 1C,D). From the field FER data, the proportion of resistance-improved progenies (in which FER was lower than that of the maize parent) in the BP and ZP populations was higher than that in the BD population (Figure 2), indicating that *Z. parviglumis* may have an improved FER resistance than *Z. diploperennis* in the B73 and Zheng58 background. This was consistent with the lack of correlation between the leaf and ear for *F. verticillioides* resistance (Figure 3). The ears of all three populations were relatively small, which may result in artificial estimates of the proportion of diseased area being smaller than the real proportion of diseased area, leading to a smaller shift in the overall FER. Therefore, the estimation bias of the proportion of diseased area may also partly contribute to the relative low FER value.

Heritabilities observed across environments in this study are consistent with estimates from previous reports (Robertson-Hoyt et al., 2006; Zila et al., 2013; Chen et al., 2016). We found that in the BD and ZP populations, FER resistance was negatively and positively correlated with flowering time (DTA and DTS) and yield (SYPP and SPPM), respectively (Figures 4A,C). As inoculation was performed at the same time for each population, late-flowering plants provided more time for *F. verticillioides* growth before seed maturation, thus explaining the negative correlation of FER resistance with flowering time. In contrast, the positive correlation with yield was due to yield-determining QTLs located closely with *qFERs* (*qFER*bd5.177, *qFERbd*10.140, and *qFER*zp4.066), and the effect of the same allele on FER resistance and yield was consistent at these QTLs (Figure 5; Table 4; Supplementary Table S5). High yield and immunity toward pathogens are important objectives in plant breeding; however, immunity often comes with yield penalties (Ning et al., 2017). Although many reports have provided new knowledge and novel strategies to minimize the costs of resistance, it remains difficult to develop new crop cultivars with strong, durable disease resistance and low yield penalty in the field (Ning et al., 2017). *TBF1* is an important transcription factor involved in the growth-to-defense switch upon immune induction (Pajerowska-Mukhtar et al., 2012). Recently, the immune-inducible promoter and two pathogen-responsive upstream open reading frames (uORFs) of *TBF1* were used to drive the leucine-rich repeat *SNC1* (suppressor of *NPR1*) gene in *Arabidopsis* and the *AtNPR1* (*Arabidopsis NON-EXPRESSOR OF PATHOGENESIS-RELATED GENES 1*) gene in rice (Xu et al., 2017). Surprisingly, the translational control of the two genes mediated by *TBF1* uORFs resulted in broad-spectrum disease resistance without growth penalties (Xu et al., 2017). Therefore, the resistance-yield synergistic QTLs identified in the BD and ZP populations have an important potential for resistance breeding.

Fusarium ear rot resistance QTLs have often appeared to be contradictory in different studies, probably because of the strong environmental influence and a minor effect of QTLs (Lanubile et al., 2017). Therefore, in this study, only four coincident additive QTLs (*qFER*bd5.177, *qFER*bd10.140, *qFER*zp4.066, and *qFER*zp5.116) were identified in the BD and ZP populations in two environments, as the population size was small and most of the FER QTLs had a low PVE (Supplementary Table S5; Table 3). Four epistatic *qFER*s were identified in the BP population (Supplementary Figure S4), which may be the reason why we did not identify additive *qFER*s in this population. Several previous reported candidate loci and genes associated with FER were found to be located in or near *qFER*bd5.177, *qFER*bd10.140, or *qFER*zp4.066. Based on three previous reports, candidate genes GRMZM2G145594 (which appeared in two reports), GRMZM5G857701, and GRMZM2G154628 are very close to *qFER*bd5.177 (Ju et al., 2017; Han et al., 2018; Stagnati et al., 2019). A GWAS revealed that GRMZM2G018353 and GRMZM2G005633 are very close to *qFER*bd10.140 (Stagnati et al., 2019). A gibberella stalk rot related QTL (*qgsr3*) is close to *qFER*zp4.066 (Ueguchi et al., 1993). However, no reported FER-related loci or genes were found near *qFER*zp5.116, hence we consider it a novel QTL. Four *F. verticillioides* inducible genes have been identified in *qFER*bd10.140. A large number of homologs of three of the four genes have been reported to be involved in plant disease resistance (Walters, 2003; Huang et al., 2021; Zhang and Sun, 2021).

## Conclusion

In this study, we found no correlation between the resistance to *F. verticillioides* in the leaf and ear. Two highly reliable resistance-yield synergistic QTLs (*qFER*bd10.140 and *qFER*zp4.066) were identified in the BD and ZP populations, which may be of importance for resistance breeding.

## Data Availability Statement

The data presented in the study are deposited in the NCBI repository with accession number PRJNA857178. The datasets (genotype and phenotype) presented in this study can also be found in Supplementary Tables S2–S4.

## Author Contributions

XF designed the research study. HX, DZ, XF, XX, and QW performed the main part of experiment. XF, HX, and YL analyzed the data and wrote the article. FW, JX, and YL are responsible for managing the materials. All authors read and approved of its content.

## Funding

This research was funded by the National Key Research and Development Program of China (2021YFD1200700), the Key Research Program of the Department of Science and Technology of Sichuan province, China (2021YFYZ0017, 2021YFH0053, and 2020YFH0116), and the National Natural Science Foundation of China (31871640, 32072074, and 31901557).

## Conflict of Interest

The authors declare that the research was conducted in the absence of any commercial or financial relationships that could be construed as a potential conflict of interest.

## Publisher's Note

All claims expressed in this article are solely those of the authors and do not necessarily represent those of their affiliated organizations, or those of the publisher, the editors and the reviewers. Any product that may be evaluated in this article, or claim that may be made by its manufacturer, is not guaranteed or endorsed by the publisher.
