# Supplementary material for: Identification of Fusarium verticillioides Resistance Alleles in Three Maize Populations With Teosinte Gene Introgression
Source: Front Plant Sci. 2022 Jul 14;13:942397. doi: 10.3389/fpls.2022.942397 (PMC9331921; doi:10.3389/fpls.2022.942397)
Supplement: Supplementary file 1 [file Data_Sheet_1.docx]

Supplementary Material

# Supplementary Figures


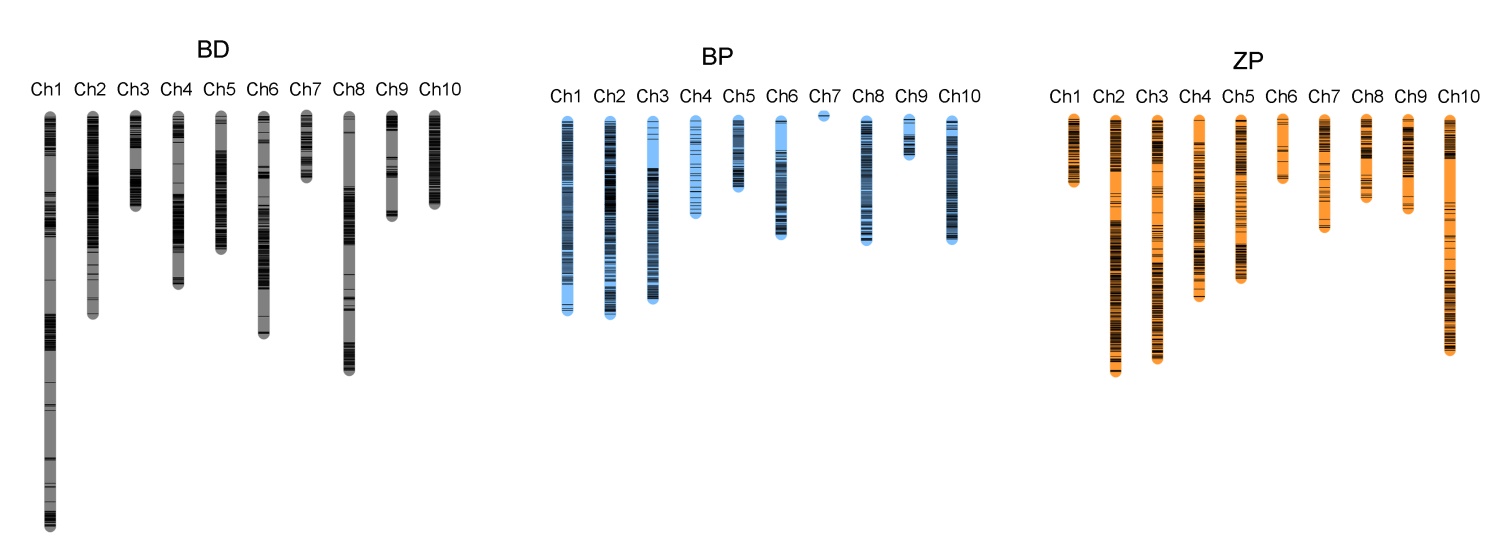


**Figure S1. Genetic maps of three populations constructed by using bins as the molecular markers.**


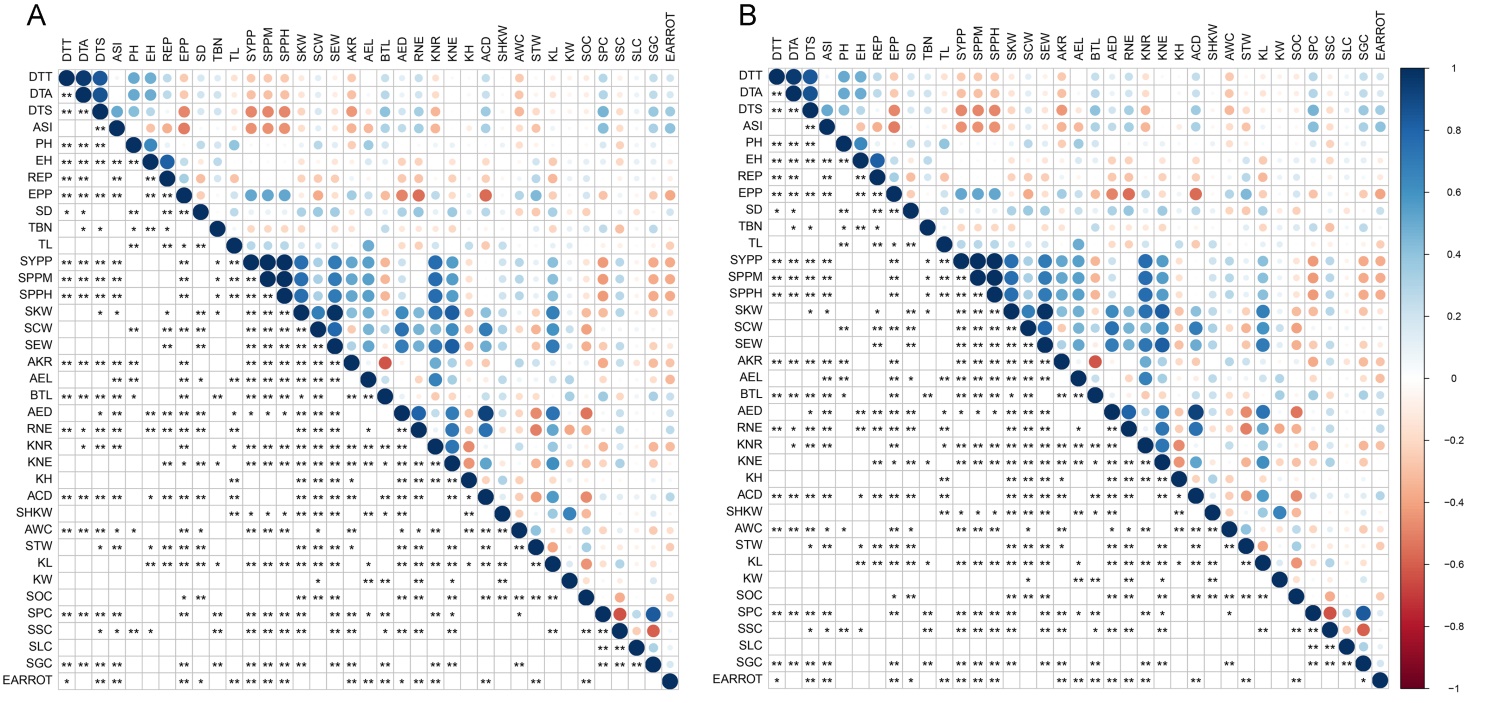


**Figure S2. Correlation between FER resistance and other agronomic traits in BD population.** FER was calculated based on (A) the proportion of diseased area, and (B) the absolute diseased area. The BLUP value of FER from 2021 was used. The relative coefficient was represented by the area and color of filled circle labeled from dark red to dark blue in the right upper part of a panel. Significance was displayed as asterisk in the left lower part of a panel. **P*  < 0.05, ***P*  < 0.01.


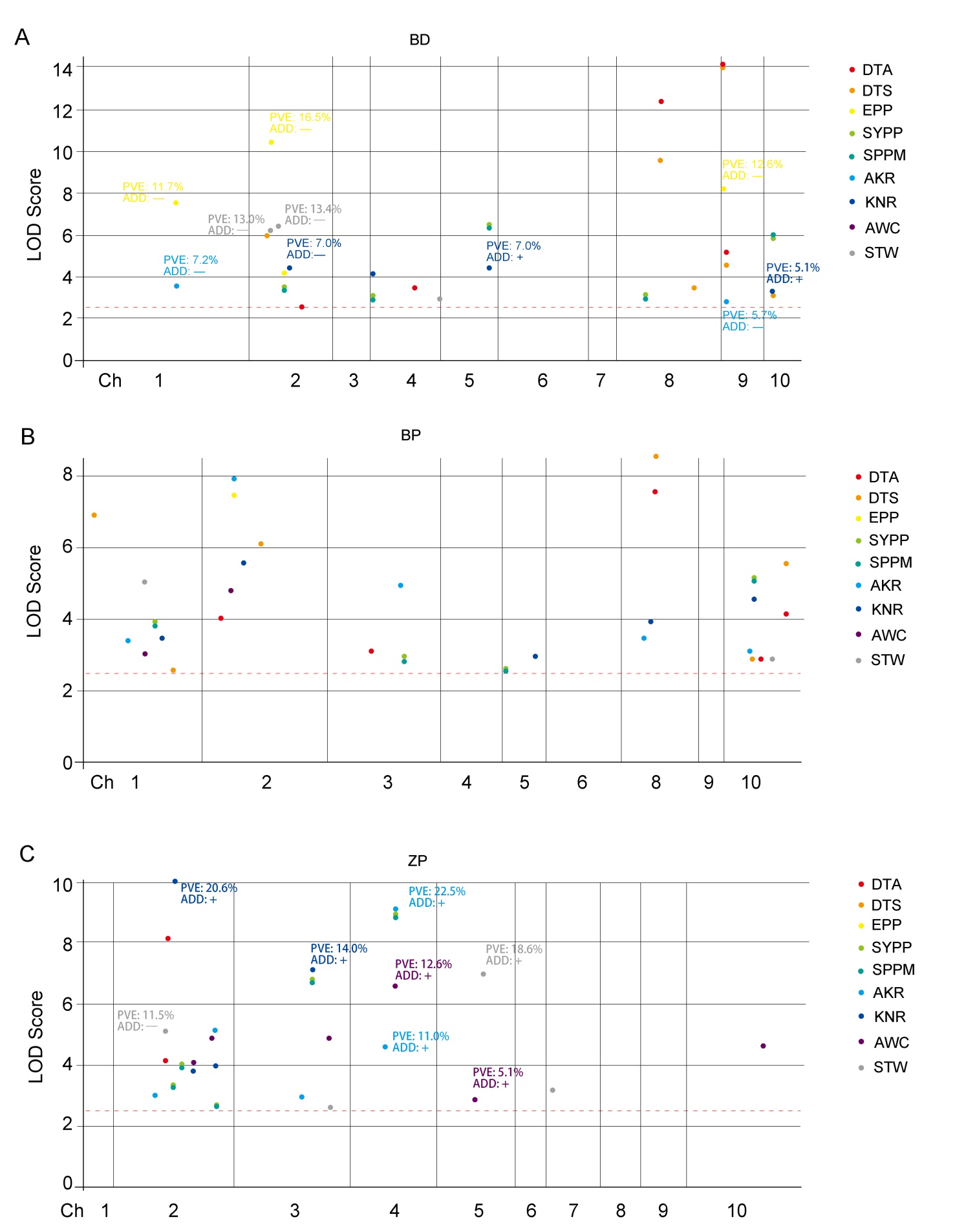


**Figure S3. Mapping for additive QTLs for FER-correlated traits in three populations.** The BLUP values of each trait from 15 replicates in five environments were used for QTL mapping. The red dotted lines indicate the LOD threshold, and QTLs with higher LOD than this threshold are considered as candidate QTLs.DTA: day to anthesis, DTS: day to silking, EPP: ear number per plant, SYPP: standard yield per plant, SPPM: standard production per Mu, AKR: average kernel rate, KNR: kernel number per row, AWC: average water content, STW: standard test weight. PVE: phenotypic variation explained by this QTL. ADD: the estimated additive effect of maize parent at this QTL.


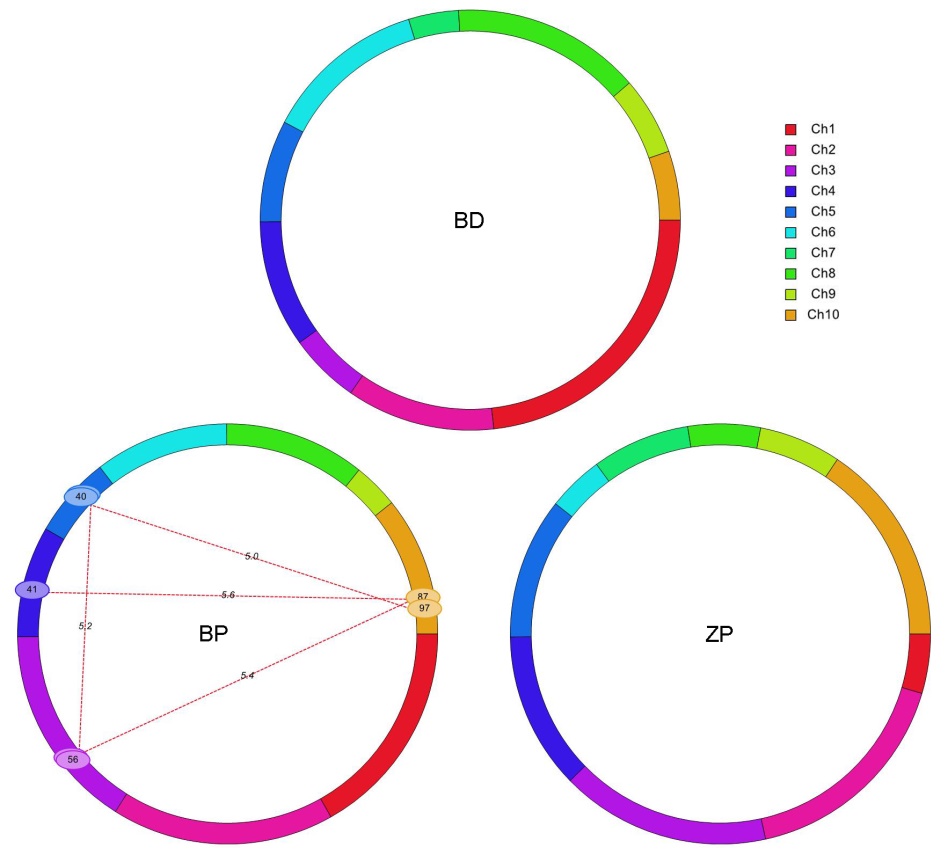


**Figure S4. Mapping for epistatic QTLs in three populations.** The BLUP value of FER from six replicates in two environments was used for QTL mapping. The numbers on chromosomes represent the genetic position of a locus. The numbers on dotted lines represent the LOD value
